# Conversion surgery for advanced hepatocellular carcinoma after combination treatment of lenvatinib and camrelizumab: a case report

**DOI:** 10.1186/s12957-023-02910-4

**Published:** 2023-01-31

**Authors:** Zhihong Chen, Zhenrong Chen, Wu Fan, Yiping Zou, Yuanpeng Zhang, Ning Shi, Haosheng Jin

**Affiliations:** 1 Department of General Surgery, Guangdong Provincial People’s Hospital, Guangdong Academy of Medical Sciences, Southern Medical University, Guangzhou, China; 2 Shantou University Medical College, Shantou, China; 3 Guangdong Cardiovascular Institute, Guangdong Provincial People’s Hospital, Guangdong Academy of Medical Sciences, Guangzhou, China

**Keywords:** Hepatocellular carcinoma, Conversion hepatectomy, Lenvatinib, Camrelizumab, Case report

## Abstract

**Background:**

Hepatocellular carcinoma (HCC) is an aggressive malignancy with high morbidity and mortality. Conversion therapy can improve surgical resection rate and prolong survival time for patients with advanced HCC. We show that combination therapy with lenvatinib and camrelizumab is a novel approach to downstage unresectable HCC.

**Case presentation:**

A 49-year-old man was diagnosed with massive HCC with hilar lymph node and lung metastases. Since radical resection was not feasible, lenvatinib and camrelizumab were administered as first-line therapy. After 10 cycles of camrelizumab and continuous oral administration of lenvatinib, the tumor exhibited striking shrinkage in volume indicating a partial radiological response, accompanied by a reduction in the alpha-fetoprotein levels, followed by salvage resection. Intriguingly, an improvement in predictive biomarkers, like lactate dehydrogenase (LDH) and neutrophil-to-lymphocyte ratio (NLR), was observed. Notably, the pathological examination found high levels of necrosis in the resected tumor, and flow cytometry analysis indicated a significant increase in the ratio of CD5+ and CD5− B lymphocytes in the peripheral blood. After the treatment, the overall survival period was over 24 months, and no recurrence was observed 17-month post-surgery.

**Conclusions:**

A combination of lenvatinib and camrelizumab may be a new conversion therapy for initially unresectable HCC to resectable HCC, thus contributing to improve the disease prognosis. In addition, the combination regimen could cause an activated immune response, and LDH, NLR, and CD5+ B-cell levels might be predictors for immunotherapy efficacy.

## Background

Hepatocellular carcinoma (HCC) is one of the most malignant cancers that seriously impair human health and life. It accounts for 75–90% of liver cancers and rank sixth and fourth worldwide in morbidity and mortality, respectively [1]. Routine treatment of HCC includes hepatectomy, interventional therapy, chemotherapy, radiotherapy, and other locoregional treatment [2]. For early-stage HCC, radical resection is a preferred option that significantly improves survival prognosis; however, most patients are diagnosed at an advanced stage.

Patients with unresectable HCC could undergo downstaging to reduce the tumor volume and will be eligible for salvage hepatectomy or liver transplantation. In addition to reducing the tumor volume, downstaging also helps in decreasing metastases and portal vein tumor thrombus and increasing residual liver volume through certain treatments [3, 4]. The traditional conversion methods include transcatheter arterial chemoembolization (TACE), portal vein embolization (PVE), transarterial radioembolization (TARE), radiofrequency ablation (RFA), percutaneous ethanol injection (PEI), associating liver partition and portal vein ligation for staged hepatectomy (ALPPS), and so on.

In recent years, immunotherapy and molecular targeted therapy have become new treatment modalities for intermediate or advanced-stage HCC, which have significantly progressed and achieved favorable conversion rates [5].

For instance, lenvatinib, a tyrosine kinase receptor inhibitor (TKI), was approved as a first-line treatment for advanced HCC after sorafenib in 2018. Lenvatinib acts on vascular endothelial growth factor (VEGF) receptors 1–3, fibroblast growth factor (FGF) receptors 1–4, platelet-derived growth factor (PDGF) receptor α, RET, and KIT [6, 7]. REFLECT, an open-label, phase 3, multicenter, non-inferiority clinical trial showed that lenvatinib-treated patients had a non-inferiority improvement in overall survival (OS) compared to sorafenib (13.6 vs. 12.3 months, *HR*: 0.92, 95% *CI*: 0.79–1.06) [8]. In addition, the progression-free survival time (PFS) was significantly improved in lenvatinib-treated patients (7.4 vs. 3.7 months, *HR*: 0.66, 95% *CI*: 0.57–0.77), and the treatment outcome of patients from Asia Pacific region treated with lenvatinib was better than those treated with sorafenib [9].

Camrelizumab is a programmed cell death factor-1 (PD-1) immune checkpoint inhibitor (ICI) that blocks the PD-1/programmed cell death factor receptor ligand-1 (PD-L1) pathway and activates the ability of the immune system to recognize and kill cancer cells. As the first approved domestic drug for the second-line treatment of HCC in China, camrelizumab has entered the 2020 edition of Chinese Society of Clinical Oncology (CSCO) guidelines for primary liver cancer and received an expert recommendation (level 2A evidence) [10]. Of note, camrelizumab showed promising treatment outcomes in clinical trials. For instance, a phase 2 monotherapy clinical trial (NCT02989922) showed that the overall objective response rate (ORR) for camrelizumab treatment was 13.8%, and a 6-month OS rate was 74.4% during the median follow-up period of 12.5 months [11]. The phase 2 clinical trial of combination therapy with apatinib (RESCUE) showed that the ORR of first-line treatment was 34.3%, the disease control rate (DCR) was 77.1%, the median PFS was 5.5 months, and the median OS was not yet mature. Furthermore, the 9-month OS rate was 86.7%, the 12-month OS rate was 74.7%, and the 18-month OS rate was 58.1%, making the combination of camrelizumab and apatinib a novel first-line treatment for HCC [12].

Even though ICI therapies are approved for the treatment of HCC, most studies have shown that ICI monotherapy alone cannot achieve the desired treatment efficacy [13]. Hence, the combination of ICI and TKI may be desirable as they can modulate the tumor immune microenvironment and promote the therapeutic effect of ICI on tumors. Furthermore, unlike apatinib, lenvatinib is shown to block FGFR4, decrease tumor PD-L1 expression, restrict Treg differentiation, and thereby contribute to anti-PD-1 efficacy [14, 15]. Previous studies have shown that lenvatinib plus camrelizumab is effective and safe for advanced HCC that had progressed after treatment. However, case reports evaluating the first-line combination of lenvatinib and camrelizumab in surgery after HCC conversion therapy are generally lacking [16, 17]. In this case study, the first line combined therapy-induced partial response (PR) for the lesions, and the intrahepatic lesions were successfully removed by hepatectomy after downstaging. Predictive biomarkers can assist clinicians in evaluating and weighing the potential benefits and risks of specific treatments, thereby screening out those who respond to treatment and guiding further treatment [18]. The changes in laboratory test results were used to explore effective markers for predicting efficacy.

## Case presentation

A 49-year-old Chinese man with chronic hepatitis B virus (HBV) infection for more than 10 years was admitted to our hospital to treat an intrahepatic lesion. The man went to the local hospital to investigate dark yellow urine 18 months ago. Contrast-enhanced computed tomography (CT) imaging examination indicated a massive tumor in the right hepatic lobe with multiple intrahepatic foci surrounding it. The tumor was about 71 mm × 55 mm in size, accompanied by multiple lymph node enlargements near the inferior vena cava and multiple lung metastases. The positron emission tomography/magnetic resonance imaging (PET/MRI) images before admission also revealed similar signs. Since the tumor was unresectable and showed a Child-Pugh score of 6, grade A, with an Eastern Cooperative Oncology Group Performance Score (ECOG-PS) of 0, the patient received immunotherapy combined with targeted therapy. The regimen consisted of lenvatinib (12 mg, po, qd) and camrelizumab (200 mg, ivdrip, q3w). In addition, propofol tenofovir for treating chronic hepatitis B was orally administered to control HBV DNA replication.

**Fig. 1:**
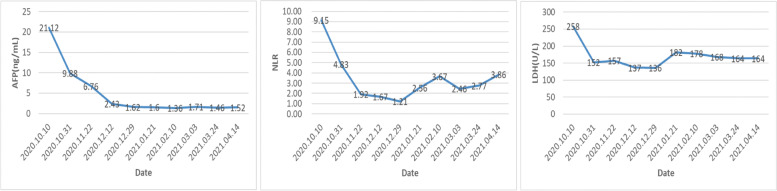
The preoperative change of the patient’s serum AFP, LDH, and NLR from October 10, 2020, to April 14, 2021. AFP, alpha-fetoprotein; LDH, lactate dehydrogenase; NLR, ratio of neutrophils to lymphocytes

**Fig. 2:**
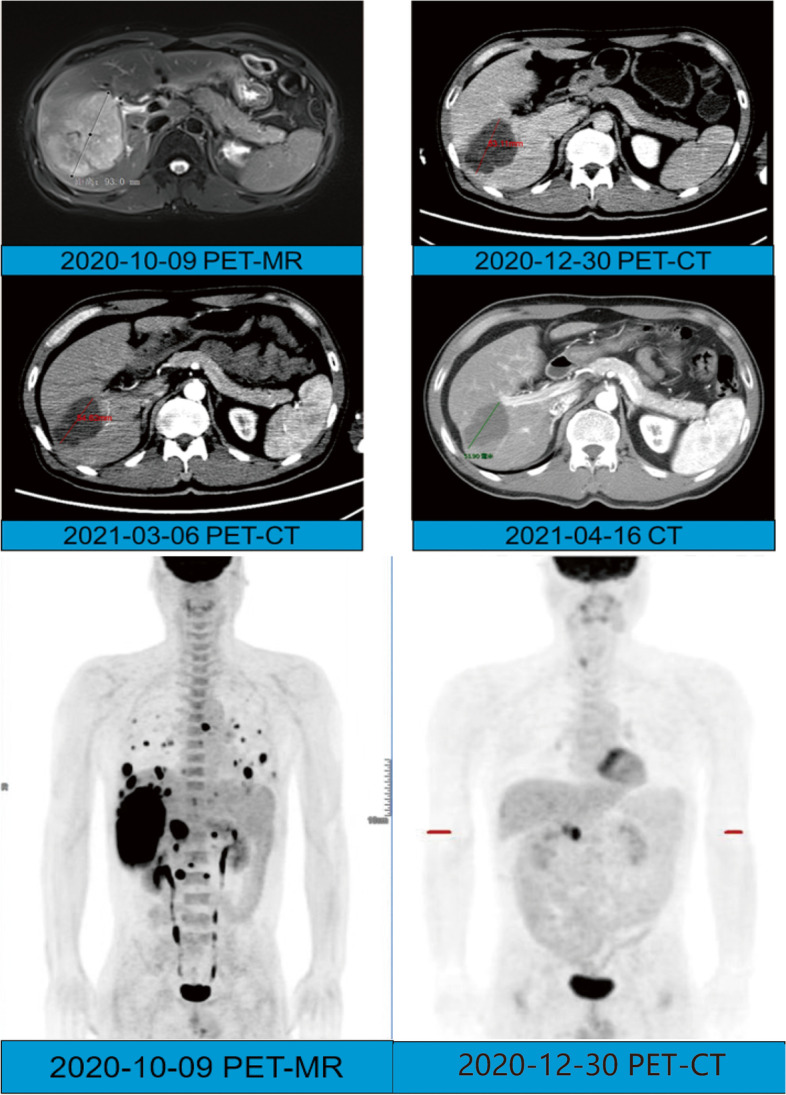
The preoperative imaging of the patient’s intrahepatic lesion from October 9, 2020, to April 16, 2021

**Fig. 3:**
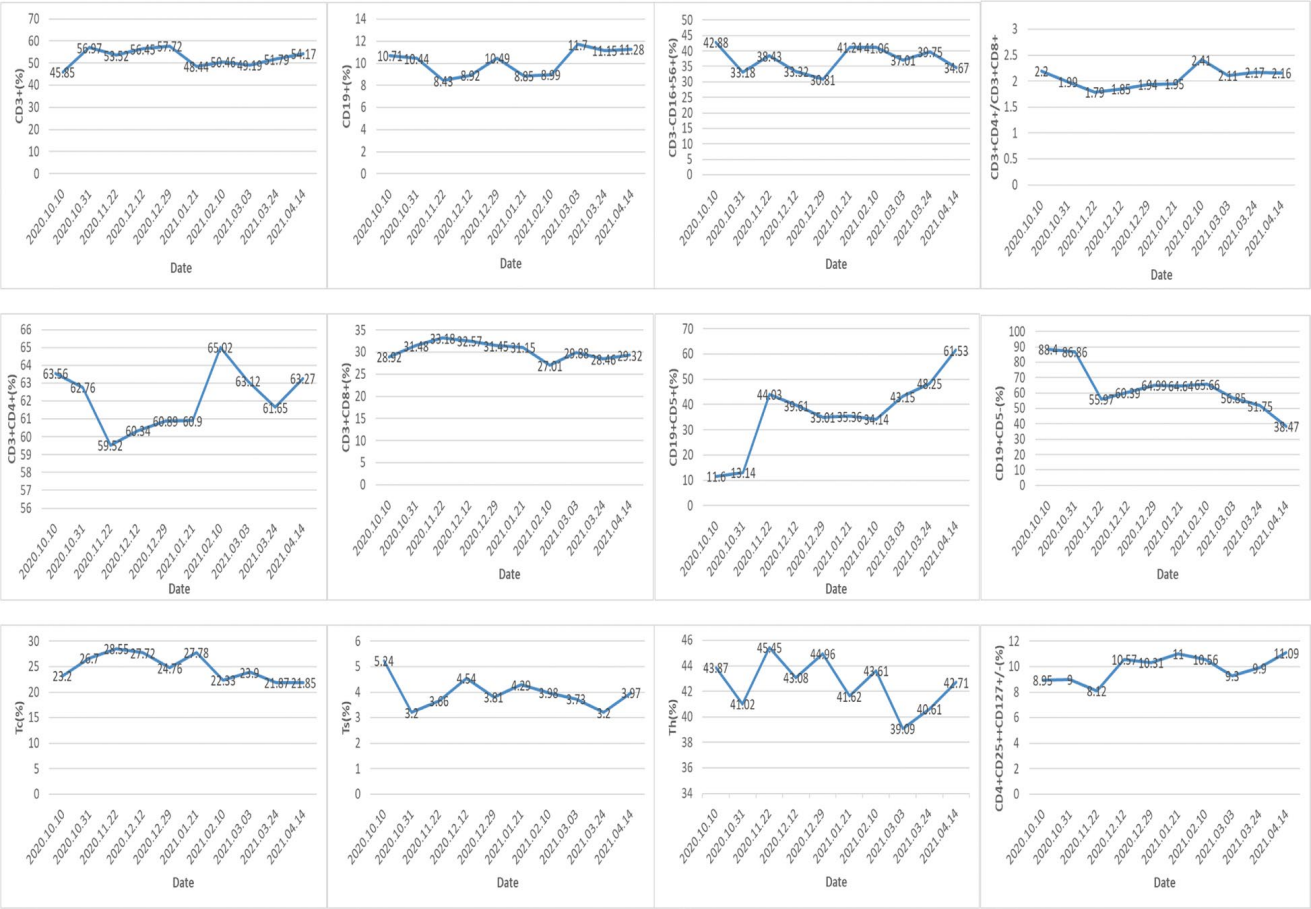
The preoperative change of the patient’s peripheral blood lymphocyte subgroups from October 10, 2020, to April 14, 2021. Tc, cytotoxic T cell; Ts, suppressor T cell; Th, helper T cell

The patient completed 10 courses of ICIs from October 10, 2020, to April 14, 2021. During the treatment course, the only adverse event experienced by the patient was a hand-foot skin reaction, a mild adverse reaction that could gradually disappear. As a result of the treatment, the alpha-fetoprotein (AFP) level dropped to the normal range. Additionally, the levels of neutrophil-to-lymphocyte ratio (NLR) and lactate dehydrogenase (LDH) showed a similar downward trend (Fig. 1). Furthermore, based on imaging examination, a shrinkage in the tumor size was observed and was evaluated as partial response (PR) according to the response evaluation criteria in solid tumors (RECIST) criterion (Fig. 2) [19]. Simultaneously, before each immunotherapy session, we also analyzed the lymphocyte subgroups in peripheral blood by flow cytometry. We found that the proportion of T cells was lower than the normal range, while the proportion of NK cells was higher. Besides, a mild increase in CD5-positive B lymphocytes was observed, while the CD5-negative B lymphocytes showed a decrease. However, the proportions of other lymphocytes did not change significantly during the treatment course (Fig. 3).

**Fig. 4:**
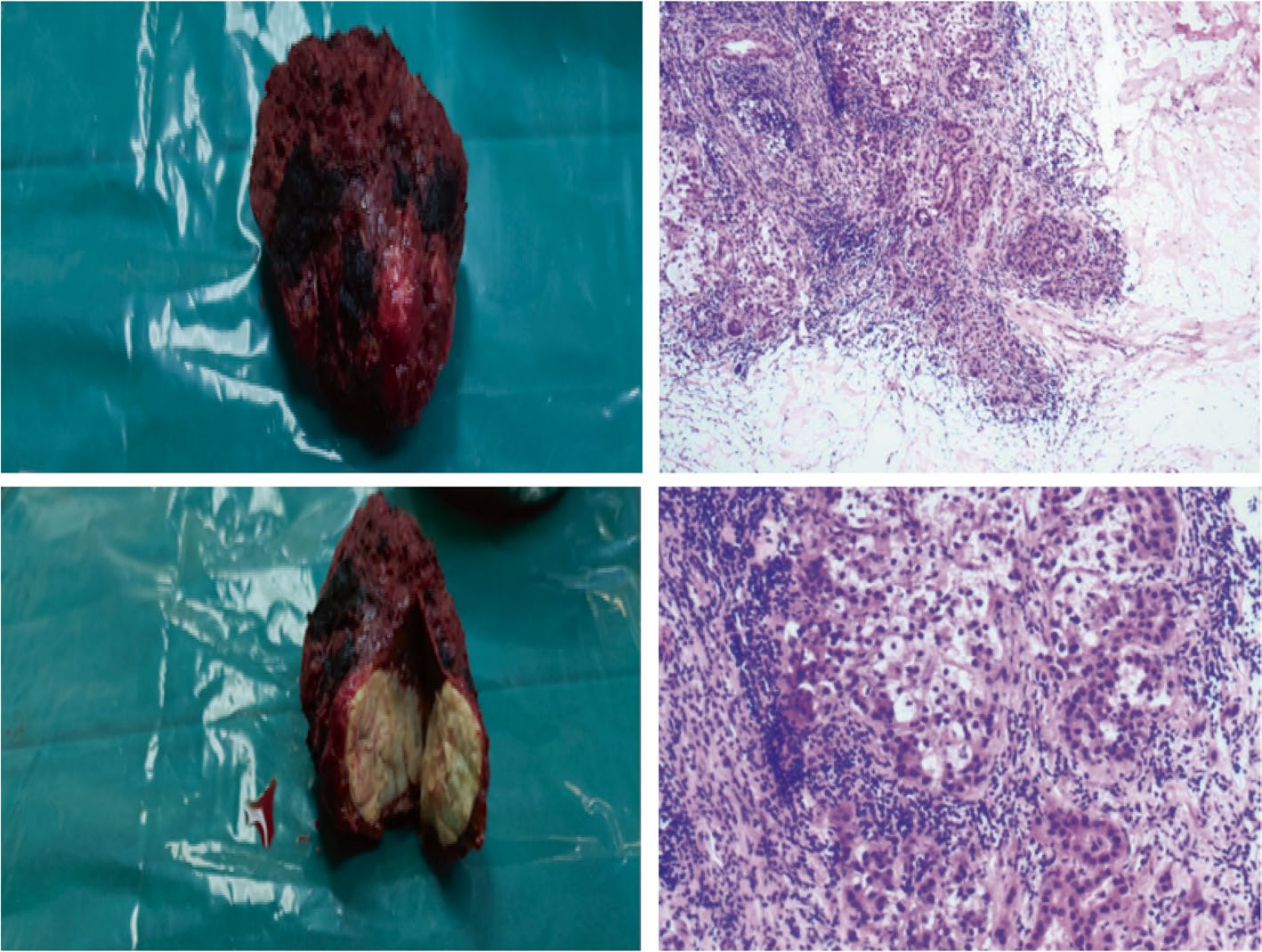
The macroscopic and microscopic views of the patient’s intrahepatic lesion

The patient underwent partial hepatectomy 7 days after the end of the 10th course of camrelizumab and the discontinuation of oral lenvatinib. Postoperative pathological results indicated that the resected liver segment 5/6 tumor was HCC, grade 2, with posttreatment response, large necrotic area, and negative resection margin (Fig. 4). After postoperative pain relief, albumin supplementation, and other treatments, the patient’s liver function improved, and the levels of liver enzymes, albumin, and bilirubin returned to the normal range. After the operation, the patient had mild bile leakage and recovered without clinical intervention. No other complications occurred.

Our finding show that the patient’s intrahepatic lesion was successfully resected, and the imaging examination suggested enlarged hilar lymph nodes and decreased glucose metabolism of the pulmonary metastatic lesion. However, he was still advised to continue to lenvatinib and camrelizumab combination as adjuvant therapy from the 18th-day post-operation. Since January 19, 2022, the FOLFOX4 regimen has been added to the adjuvant therapy regimen to enhance the efficacy and to improve prognosis. Eleven months after the operation, the tumor indicators were still normal, and the patient was in good condition and did not complain of discomfort. The overall survival time was more than 24 months, and there was no recurrence in the postoperative 17 months.

The patient had signed the informed consent, and approval from the hospital ethics committee was obtained (no. KY-Q-2021-084-02). The patient had agreed to report his images and other clinical information in the journal. He understands that his name will not be published, and due efforts will be made to conceal his identity.

## Discussion and conclusions

Most HCC patients are diagnosed at an advanced stage, and systemic therapy, especially the combination of anti-PD1 antibodies and anti-angiogenesis agents, is recommended because of their favorable therapeutic effect [20]. Since liver resection, even with extrahepatic metastases, significantly improves the survival outcome of HCC [21], patients with HCC may undergo surgery for a better prognosis. Hence, it is critical for patients with unresectable tumors to undergo successful conversion therapy for future surgical resection of the tumor.

Numerous case reports have described the efficacy and safety of the combination of immunotherapy and molecular-targeted therapy for HCC [22]. In this study, we reported a case in which conversion therapy for initially unresectable HCC was successfully achieved following the combination treatment of lenvatinib and camrelizumab. After searching the ClinicalTrials.gov database, we found several clinical trials that used lenvatinib and camrelizumab combination for advanced HCC, including NCT04443309, NCT04627012, and NCT04639284 [23, 24]. However, it is essential to initiate more clinical trials to evaluate the efficacy, safety, and conversion rate of this novel combination regimen for HCC and to investigate suitable markers to identify the target population.

Our case report confirmed the critical role of the ICI and TKI combination regimen in the conversion therapy of initially unresectable advanced HCC. The VEGF-VEGFRs signaling pathway is shown to participate in the regulation of the tumor immune microenvironment in several ways:

1. VEGF can prevent the maturation of antigen-presenting cells, such as dendritic cells, and reduce the activation of T lymphocytes.
2. VEGF can promote the formation of dysfunctional tumor vasculature, thereby blocking T-lymphocyte infiltration in tumor tissues and triggering T-lymphocyte death.
3. VEGF stimulates the amplification of myeloid-derived suppressor cells, resulting in the immunosuppressive tumor microenvironment and weakening the anti-tumor immune response.

Therefore, inhibition of the VEGF-VEGFRs pathway may facilitate the normalization of the tumor vasculature, increase the infiltration of immune cells and anticancer drugs, and thus potentially improve the therapeutic efficacy [25].

In addition to the improvement in tumor size, the biomarkers of the patients also showed considerable improvement. Notably, the AFP levels of the patient gradually decreased to the normal range as the treatment progressed, indicating that the progression of HCC was attenuated and the treatment was effective. Similar trends were also observed for NLR and LDH. A previous study has shown that NLR can be used as a biomarker to predict the efficacy of PD-1 inhibitors in HCC [26]. Furthermore, numerous studies have mentioned that the LDH level is associated with the prognosis of HCC [27]. LDH is an important enzyme involved in tumor glycolysis and facilitates energy production and angiogenesis. Therefore, increased LDH could promote tumor growth and invasion. Decreased LDH concentration is shown to predict the efficacy of sorafenib, indicating it may also be associated with the treatment efficacy of combination therapy [28]. Previous studies have shown that the lung immune prognosis index (LIPI) integrated by NLR and LDH is of great significance for predicting the efficacy of ICIs-treated non-small cell lung cancer patients. LIPI was also associated with survival outcomes in patients with advanced HCC treated with PD-1 inhibitors [29]. The detection of these biomarkers is cost-effective, simple, and rapid. Besides, alanine aminotransferase, aspartate aminotransferase, and albumin-bilirubin grades may predict the efficacy of TKIs other than sorafenib, while dysregulated TERT, CTNNB1, TP53 FGF19, and TP53 may be associated with ICI efficacy [30]. The ratio of CD5+ and CD5− B cells indicated that the inhibition of the PD-1/PD-L1signaling pathway might have induced the activation of B lymphocytes in the peripheral blood. Moreover, CD5− B lymphocytes could be induced to differentiate into the CD5+ B lymphocytes which perform the functions like antibody production, immune response, and immune homeostasis. Recent studies suggested that CD5 played an unfavorable role in the modulation of various immune responses and hence could be a therapeutic target for different pathologies, including autoimmune diseases, cancers, and infections [31].

As a multi-target TKI, lenvatinib inhibits several signaling pathways associated with tumor development, such as VEGF/VEGFR, FGF/FGFR, and RET signaling pathways. It has demonstrated a strong anti-tumor effect in vitro and in vivo. REFLECT trial has demonstrated the non-inferiority of lenvatinib is to that of sorafenib. In addition, it was found in animal models that lenvatinib has immunomodulatory activity and can reduce the proportion of monocytes and macrophages and increase the proportion of CD8+ T cells. These effects may contribute to the anti-tumor activity of lenvatinib monotherapy and dual therapy with PD-1 inhibitors. Therefore, further investigation is required to better understand the mechanistic effects of the combination of lenvatinib plus PD-1 inhibitors in the treatment of advanced HCC [32]. Camrelizumab is different from other PD-1 inhibitors in terms of binding mode and affinity [33]. Camrelizumab and levatinib have achieved favorable results in clinical trials conducted among the Chinese population and have been approved for marketing in China. After being covered by national medical insurance, it is inexpensive compared to the combination of bevacizumab and atezolizumab [17]. The case report further reveals that lenvatinib combined with camrelizumab is a feasible conversion approach. Continuous use of TKIs can avoid delayed effects or failure of immunotherapy; also, it helps regulate the tumor immune microenvironment, promote antigen exposure and presentation, and improve efficacy. Of note, patients who received surgical treatment after translational therapy appeared to have better outcomes compared to their nonsurgical treatment counterparts. However, previous studies have shown low conversion rate for the combination of PD-1 inhibitors and TKIs [34]. Besides, the appropriate timing of surgery and the choice of postoperative adjuvant treatment for the patients after conversion therapy are still unclear, and more clinical trials are urgently needed to address these questions.

In conclusion, we report a case of advanced HCC who underwent successful hepatectomy without positive margins after conversion with lenvatinib and camrelizumab combination therapy. It may be a new way for the downstaging of unresectable intermediate-advanced HCC and help improve patients’ prognoses. In addition, our results suggest that the combination regimen may apply to patients with higher levels of LDH and NLR and a lower proportion of CD5+ B cells.

## Data Availability

The data and images of the patient are contained in the medical record system of Guangdong Provincial People’s Hospital. In addition, the data supporting the conclusions of this article are included within the manuscript, figures, and tables.

## Abbreviations

HCC: Hepatocellular carcinoma
TACE: Transcatheter arterial chemoembolization
PVE: Portal vein embolization
TARE: Transarterial radioembolization
RFA: Radiofrequency ablation
PEI: Percutaneous ethanol injection
ALPPS: Associating liver partition and portal vein ligation for staged hepatectomy
TKI: Tyrosine kinase receptor inhibitor
VEGF: Vascular endothelial growth factor
FGF: Fibroblast growth factor
PDGF: Platelet-derived growth factor
PD-1: Programmed cell death factor-1
ICI: Immune checkpoint inhibitor
PD-L1: Programmed cell death factor receptor ligand-1
CSCO: Chinese Society of Clinical Oncology
ORR: Objective response rate
DCR: Disease control rate
PR: Partial response
HBV: Hepatitis B virus
CT: Computed tomography
PET/MRI: Positron emission tomography/magnetic resonance imaging
AFP: Alpha-fetoprotein
NLR: Neutrophil-to-lymphocyte ratio
LDH: Lactate dehydrogenase
PR: Partial remission
RECIST: Response evaluation criteria in solid tumors
LIPI: Lung immune prognosis index
